# Student centered curricular elements are associated with a healthier educational environment and lower depressive symptoms in medical students

**DOI:** 10.1186/1472-6920-14-192

**Published:** 2014-09-17

**Authors:** Eiad Abdelmohsen AlFaris, Naghma Naeem, Farhana Irfan, Riaz Qureshi, Cees van der Vleuten

**Affiliations:** King Saud University Chair for Medical Education Research and Development, King Saud University, Riyadh, Kingdom of Saudi Arabia

**Keywords:** Curriculum, Educational environment, Depressive symptoms, Medical students

## Abstract

**Background:**

Any curriculum change is essentially an environmental change; therefore there is a need to assess the impact of any change in the curriculum on the students’ perception of the Educational Environment (EE) and psychological well-being. The objectives of the current study are to (i) compare the EE perceptions of medical students studying in a System Based Curriculum (SBC) with those studying in a traditional curriculum (ii) compare the rate of depressive symptoms among the same students studying in both types of curricula (iii) determine whether there is a difference in the EE perception and depressive symptoms based on gender and year of study.

**Methods:**

A cross sectional survey was conducted in a Saudi Medical School from 2007-2011, a period in which the school transitioned from a traditional to a SBC. A bilingual version of the Dundee Ready Educational Environment Measure (DREEM) inventory was used for measuring the EE; the Beck Depression Inventory (BDI II) was used for screening of depressive symptoms. A separate demographic questionnaire was also used. Mean scores and percentages were calculated. Continuous variables were summarized as means and standard deviation. For comparison of means, the effect size and student t test (with significance level of <0.05) were used. The percentages of the categorical data were compared using chi square test.

**Results:**

The mean total DREEM score of positive perception of the EE in the SBC students was significantly higher (better) than the traditional curriculum students (p < 0.01) with an effect size of 0.472. The mean total score on the BDI-II inventory for depressive symptoms was higher (sicker) 21.3 among the female traditional curriculum students than 16.7 among the male traditional curriculum students and the difference was statistically significant (p = 0.001). The BDI score of the female SBC students (14.7) was significantly lower (healthier) than the female traditional curriculum students (21.3). No similar change was noted for the male students.

**Conclusion:**

The current study adds to the advantages of the SBC indicating not only healthier EE for both genders but also healthier emotional well-being for female students only.

**Electronic supplementary material:**

The online version of this article (doi:10.1186/1472-6920-14-192) contains supplementary material, which is available to authorized users.

## Background

The Educational Environment (EE) refers to the social, psychological and pedagogical contexts in which learning occurs and which affect student achievements, attitudes and wellbeing [1–3]. It plays a significant role in relation to the student’s behavior, academic development and well-being. The literature suggests that although the concept is rather intangible, the effects of the EE are substantial, real and influential [2, 4].

Traditionally, curricula at most medical colleges were non-integrated, teacher-centered, information gathering, opportunistic, discipline and hospital-based with a set of mandated courses [5]. However, with changes in clinical practice and societal demands, there is a move towards more integrated curricula. The new curricula are integrated around either systems or clinical presentations [6]. The systems-based curricula (SBC) are integrated around organ systems with inclusion of early clinical and practical skills training and small-group learning [6].

Medical education is perceived as being stressful and can have a negative impact on cognitive functioning and learning [7]. Rates of depression and suicide are higher among medical students than other university studies’ students [8, 9]. The rates remain elevated even when these students become physicians.

Research studies using a variety of instruments have reported a range of figures for prevalence of depressive symptoms among medical students - 43.89% [10], 25% [11], 21.7% [12], 71.25% [13], 12-30% [14], 18.6% [15]. On the other hand, among the general population, the rate of 3% to 4% was observed [16, 17].

A study among Saudi medical students found a 57% prevalence of stress, with 19.6% of them having severe stress [18]. The presence and severity of depressive symptoms had a statistically significant association with early academic years (p < 0.000) and female gender (p < 0.002) [19]. A systematic review concluded that studying medicine is a time of significant psychological distress [20]. One of the factors found to be associated with higher stress and psychological illnesses was adjustment to the medical school environment [21]. Although the EE and the depressive symptoms have been studied separately in traditional and system based curricula; none of these studies have addressed both the variables together. The current study was carried out in a school that moved from a traditional curriculum to a system-based one providing an ideal opportunity to study the two variables together and to compare the two systems across time before and after the change.

The objectives of the current study are to (i) compare the EE perception of medical students studying in a SBC with those studying in a traditional curriculum (ii) compare the rate of depressive symptoms among the students studying in the two curricula (iii) determine whether there is a difference in perception of EE and depressive symptoms based on gender and year of study.

## Method

### Study setting and the curriculum

The current study was conducted in the medical school of King Saud University (KSU) which was established in 1973. The KSU curriculum until the year 2007/2008 was traditional, as defined by the General Medical Council (1993) and it was changed to SBC starting in the year 2008/2009. Like other Saudi medical schools, KSU operates on a single-gender basis (teaches the two genders separately). The intake of male students was more than double that of female students till the year 2007. After that, almost equal intake of male and female students was adopted.

### The traditional curriculum versus the SBC

The curriculum, before the change, was non-integrated, discipline based and teacher centered using didactic lectures as the main teaching strategy. The grading system was largely based on summative assessment system i.e. the grade A + being the highest, followed by B, C, and D for pass and F for fail. The new SBC is integrated horizontally with a move towards vertical integration. The first two years of the new curriculum consist of ten organ systems taught using problem-based learning (PBL) strategy. A student support system was established. The number of elective courses was increased. Students were introduced to ambulatory care early in the program and the duration of ambulatory care training was increased. The teaching strategies include small-group discussions, interactive lectures and self-directed learning. Teaching resources include study guides, an electronic learning management system, skills lab and virtual patients. The assessment is balanced between formative and summative assessment and the grading system is similar to the traditional curriculum grading system mentioned earlier.

### The outcome variables

#### Educational environment

The DREEM inventory was used to measure the perception of the EE. The DREEM was initially reported to be valid and culture free [22–25]. Although the minimal sources of evidence reduced its validity; it is still the mostly widely used questionnaire for the undergraduate curriculum [26]. It comprises of 50 items relating to a range of topics directly relevant to the educational climate [16] and is divided into five subscales (Additional file 1). The items of the inventory were scored as suggested by the authors of the inventory as follows: 4 for Strongly Agree (SA), 3 for Agree (A), 2 for Uncertain (U), 1 for Disagree (D) and 0 for Strongly Disagree (SD) [22]. The approximate guide to interpreting the DREEM results is summarized as follows: A mean overall total score of 0-50 is interpreted as very poor, 51-100 (plenty of problems), 101-150 (more positive than negative) and 151-200 (excellent).The higher the score of an item, the more positive is the students’ perception of the environment. The inventory has a maximum score of 200, which indicates the ideal EE.

### Depressive symptoms

The BDI-II Inventory was used to measure the depressive symptoms. It was selected because of its specificity at detecting depressive symptoms among college students [27, 28]. The BDI-II is a 21 item, self-report instrument, which measures the severity of depression in both adults and adolescents aged 13 years and older [28]. The BDI-II version was developed for the assessment of symptoms corresponding to the criteria for diagnosing depressive disorders listed in the American Psychiatric Association’s Diagnostic and Statistical Manual of Mental Disorders (DSM-IV, 1994). The instrument is rated on a four point scale ranging from 0-3. The scores from all items are added to get the total score for an individual. The higher the score, the more depressed the student is. A score of 0-13 is considered minimal depression, 14-19 = mild, 20-28 = moderate and 29-63 severe depression [28].

### Participants

The study participants included two cohorts of students: a) all 1^st^ and 2^nd^ year students enrolled in the College of Medicine, KSU during the year 2007/ 2008 studying in the traditional curriculum b) all 1^st^ and 2^nd^ year students enrolled in the College of Medicine, KSU during the year 2010/ 2011 studying in the SBC. No sampling method was used as the whole population was invited to participate.

### Data collection

The three questionnaires (including a separate demographic questionnaire) were administered to all the study participants during the middle of the second semester, more than two weeks before the mid-term exam. The instruments were administered at a time when the students were sufficiently acquainted with the school environment and informed verbal consent was taken with optional participation. An attached covering letter to the questionnaires included the names of the researchers and an assurance of confidentiality. It was mentioned in the covering letter that the purpose of the study was to assess the EE as the DREEM noticeably investigates the EE no further details of the study were mentioned, such as the association of the depressive symptoms with the EE to avoid bias.

### Ethics statement

The current study was approved by the Institutional Review Board of King Saud University –College of Medicine (reference no. 11/3106/IRB) dated 22/06/2011.

### Analysis

In order to address the first and third objectives, the mean total score of the DREEM and the mean score of the five domains of the inventory were calculated and compared for curricula cohorts, year of study and gender. In order to address the second and third objective, comparison of the mean BDI score was made between the two curricula cohorts, across the two academic years and the two genders. Descriptive statistics (mean, standard deviation and proportion) were used to describe the quantitative variables. Continuous variables were summarized as means and 95% confidence intervals (CI).The effect size and student t test were used for comparison. The chi square test was used for comparison between categorical variables. A p-value of <0.05 was considered as statistically significant. Data was analyzed using SPSS version 16.0.

## Results

### Demographics

The number of students who participated in the EE study from the SBC cohort was 249 and from the traditional curriculum were 458. The participants’ age ranged between 18 and 21 years. While a larger number of male than female students participated in the traditional curriculum, they were almost equal in the SBC (Table 1). The response rate of the DREEM inventory in both cohorts of students and the BDI-II for the SBC students was almost complete. On the other hand, only 65% of the students in the traditional curriculum had completely filled out the BDI-II.

**Table 1:** **The demographic characteristics of the two groups of students**

		Traditional curriculum number (%)	Systemic based curriculum number (%)	Total number
**Gender**	Male	330 (73.5)	127 (51.0)	457
	Female	119 (26.5)	122 (49.0)	241
Total		449	249	698
**Year of study**	First	239 (52.2)	122 (49.0)	361
	Second	219 (47.8)	127 (51.0)	346
Total		458	249	707

### DREEM scores in traditional curriculum versus SBC

The mean DREEM total score for the SBC students (118.5/200) was significantly higher than that of the traditional curriculum (94.6/200). The effect size (-0.47) was medium and of moderate practical importance, according to Cohen’s operational definition [29] (Table 2). The scores in all the five domains of the DREEM were higher in the SBC. First year students perceived the environment (mean total DREEM) better than second year in both curricula (Table 2).

**Table 2:** **A comparison of total DREEM scores and BDI scores by year of study**

	DREEM					BDI II				
	Mean total score					Mean total score				
	Traditional	System based	T value	P value	Effect Size	Traditional	System based	T value	P value	Effect size
1^st^ year	104.54	120.97	8.5	<0.0001	0.472	18.79 (10.8)	15.88 (9.4)	2.4	0.017	0.123
2nd year	91.65	118.38	12.3	<0.0001		16.81 (9.4)	15.37 (8.3)	1.27	0.20	
Total	94.6(21.0)	118.5 (23.5)	14.21	<0.01		18.0 (10.3)	15.6 (8.9)	2.91	0.004	

This was noted for both sexes in the traditional curriculum. There was no significant decline in the DREEM scores among the SBC students. There was a higher mean total DREEM score for the female students compared to their male counterparts for both years and in both cohorts (Table 3).

**Table 3:** **A comparison of total DREEM scores by sex and year of study**

Curriculum type	Year	Male			Female			Total			P value*
		N	Mean	SD	N	Mean	SD	N	Mean	SD	
Traditional	First	166	99.51	19.67	73	103.38	17.37	239	100.69	19.04	NS
	Second	168	86.10	21.62	51	93.86	18.44	219	87.90	21.14	**P** < **0.05**
	**Total**	**334**	**92.76**	**21.71**	**124**	**99.47**	**18.36**	**458**	**94.58**	**21.04**	**P** < **0.05**
SBC	First	57	114.89	22.31	73	123.73	23.22	130	119.85	23.16	**P** < **0.05**
	Second	79	112.38	22.23	65	123.09	24.63	144	117.22	23.87	**P** < **0.05**
	**Total**	**136**	**113.43**	**22.22**	**138**	**123.43**	**23.81**	**274**	**118.47**	**23.53**	**P** < **0.05**

*LEs improved in the Abdul Jabbar. cribed by Beck*.

*Results are based on two-sided tests assuming equal variances with significance level 0.05.

### BDI II scores in traditional versus SBC

The mean BDI-II total score for depressive symptoms was higher (sicker) for the students of the traditional curriculum (18.0) compared to the SBC (15.6) for the two years (Table 2), giving a small effect size (negligible practical importance) [29]. The prevalence of severe depressive symptoms was greater among the first year students compared with their second year counterparts for both curricula (Table 4). A statistically significant association (p = 0.001) was found between the student gender and the severity of depressive symptoms among the traditional curriculum students; being more severe among the female students (Table 4). The gender trend for more severe symptoms of depression was reversed among the students of SBC, though it did not reach a statistically significant association (p = 0.271).

**Table 4:** **Demographic characteristics versus BDI II scores and the statistical association** (**Traditional Curriculum and System Based Curricula**)

			Total No.	Minimal n (%)	Mild n (%)	Moderate n (%)	Severe n (%)	Mean total score (SD)	Chi square	P value
Traditional	Academic year	1st	176	63 (35.8)	42 (23.9)	40 (22.7)	31 (17.6)	18.79 (SD = 10.83)	2.626	.453
		2nd	118	45 (38.1)	33 (28.0)	27 (22.9)	13 (11.0)	16.81 (SD = 9.43)		
		Total	294	108 (36.7)	75 (25.5)	67 (22.7)	44 (14.9)	18.0 (SD = 10.3)		
	Gender	Male	213	91 (42.7)	55 (25.8)	38 (17.8)	29 (13.6)	16.74 (SD = 10.16)	16.827	.001
		Female	81	17 (21.0)	20 (24.7)	29 (35.8)	15 (18.5)	21.31 (SD = 10.07)		
SBC	Academic year	1st	122	59 (48.4)	30 (24.6)	24 (19.7)	9 (7.4)	15.88 (SD = 9.43)	.833	.842
		2nd	127	59 (46.5)	31 (24.4)	30 (23.6)	7 (5.5)	15.37 (SD = 8.36)		
		Total	249	118 (47.4)	61 (24.4)	54 (21.6)	16 (6.4)	15.6 (SD = 8.9)		
	Gender	Male	127	61 (48.0)	26 (20.5)	29 (22.8)	11 (8.7)	16.48 (SD = 10.05)	3.911	.271
		Female	122	57 (46.7)	35 (28.7)	25 (20.5)	5 (4.1)	14.72 (SD = 7.43)		

## Discussion

The response rate of the DREEM inventory for both cohorts of students and the BDI II for the SBC students was almost complete, which was reassuring. However, only two third of the students in the traditional curriculum had completely filled out the BDI-II. It could be that the students when filling out the three questionnaires were exhausted when they reached the last one (BDI-II).

The mean total DREEM score of the cohort of students from the SBC in the current study (118.5/200) is in the middle of the results of studies conducted among medical students worldwide [30–32]. The SBC students perceived the EE significantly better than the traditional curriculum students (95/200), which is consistent with the findings of other reports [4, 33, 34]. The possible reasons for this improvement could be that the instructional methods were more relaxed, practical and enjoyable. The introduction of a study guide with the SBC might have contributed to this improvement, as it enabled students to manage their time and learning tasks well in order to become self-regulated learners [31].

It is noteworthy that the scores of the first year students of the traditional curriculum (105/200) were higher than the scores of second year students (92/200). No similar decline was observed with the SBC students. The decline in the mean EE scores was observed for both genders in the traditional curriculum. The possible explanation is that the students studying in the traditional curriculum may have entered the school with optimism but were disappointed by the curriculum overload and the competitive environment. This may also be attributed to the fact that students have to face the limits of their cognitive capacities to achieve the required knowledge and also to adjust to significant changes in their daily routine [8, 35, 36]. This decline in the scores of all the EE domains in the second year, is consistent with that reported by other medical schools [37, 38].

The significantly higher mean total DREEM scores for female students in both the traditional (99/200), and the system based (123/200) curricula as compared to their male counterparts (93/200) and (113/200) respectively, suggests that the female students were relatively more satisfied with learning, teaching and the school atmosphere, which is consistent with the reports of other studies [30, 39, 40].

Reasons for depression among medical students are multifactorial and include adjustment to large volume of information, competition and concern over failure in their studies. In addition to the genetic and other environmental factors that occur regardless of the educational stress [19, 41]. Nurturing students’ personal development, psychological and physical health is very important and similar in importance to acquiring the knowledge and skills needed to be future physicians [42].

For SBC female students, the mean total BDI score was significantly lower than for traditional female students, but this trend was not observed with the male students. What is there about female students who seem to have benefited psychologically by the change to the SBC? There must be a phenomenon related to the female students that indicates a positive impact of curriculum change on their mental health and wellbeing. It appears that the female students prefer PBL style of learning and had better satisfaction with the EE compared with their male counterparts. As has been mentioned earlier that the curriculum at KSU operates on single gender basis and therefore, another plausible explanation might be that the female student’s teaching in the traditional curriculum may have been inferior to that of their male counterparts. It is possible that the change in the teaching strategy in the SBC e.g. small group teaching, less curricular load and smaller class size had a greater positive impact on their psychological wellbeing and perception of EE. These plausible reasons need to be confirmed by a qualitative study and deserve further exploration [42].

Several studies have reported the rate of depressive symptoms among medical students, but have not explored its relationship with their perceptions of the EE.

The lower rate and severity of depressive symptoms among second year students compared with the first year students in the current study contradicts the findings of a Turkish study which reported a significant rise in the scores on the BDI-II among medical students between the first and second years [41]. Similarly another study among Chinese medical students found doubling of depression in medical students between the beginning and the end of the first year [43].

It is interesting to note in the current study that while the first year students were more satisfied with the EE, they had a slightly higher rate of depressive symptoms than the second year students although the difference was not significant. This phenomenon might be explained by the “transactional theory of stress and coping” [44] which explains the stressed person -environment relationship.

The finding of a higher rate of depressive symptoms among female students in the traditional curriculum is consistent with findings of other studies [10, 21, 45–48]. On the other hand, the finding of a lower rate of depressive symptoms among female students in the SBC (though statistically not significant) contradicts the finding of other studies that found more female than male medical students becoming depressed during the first two years of schooling [10, 21, 47]. However, a recent study from Malaysia reports similar findings [48]. Women, in general ,are known to have a higher lifetime risk of depression and anxiety than men [20]. A multi-country study of undergraduate students reported a higher rate of depression among males than females in Saudi Arabia [49]. This study also reported that in some other regional countries females tended to have higher rates of depression than males namely Iraq, Syria, Egypt, Pakistan, Algeria, Oman, Qatar, Morocco, and Kuwait [49]. On the other hand, no significant gender differences in depression were observed in Lebanon, Tunisia, Palestine, United Arab Emirates, Yemen, Jordan, and Sudan [49].

### Limitations and strengths

The current study used comparable data for the system based and the traditional curricula. Since, few studies have addressed the impact of student centered teaching on student psychological wellbeing; the results of this study can be used as a proxy indicator of student wellbeing and can be accepted as a baseline for further investigation. This study has some limitations including the use of self-administered inventories rather than structured interviews for exploration and clinical diagnoses. Furthermore, it was conducted in only one school. The sample size was relatively large, and the response rate was high for all the study groups, however, there was a relatively low response rate for the BDI-II inventory by students of the traditional curriculum.

## Conclusion

The SBC students perceived the EE more positively than their traditional curriculum counterparts. Depressive symptoms were found to be significantly less and milder among the female SBC students compared with their male counterparts in the traditional curriculum. The current study adds to the advantages of the SBC in terms of healthier EE for both genders but an improved emotional well-being for female students only.

## Electronic supplementary material

Additional file 1: **Guide for interpretation of DREEM scores.** (PDF 93 KB)
