# Supplementary material for: Student centered curricular elements are associated with a healthier educational environment and lower depressive symptoms in medical students
Source: BMC Med Educ. 2014 Sep 17;14:192. doi: 10.1186/1472-6920-14-192 (PMC4177056; doi:10.1186/1472-6920-14-192)
Supplement: Supplementary file 1 — Additional file 1: Guide for interpretation of DREEM scores. (PDF 93 KB) [file 12909_2013_1019_MOESM1_ESM.pdf]

Appendix 1: Guide for interpretation of DREEM scores.

**DOMAIN**	**Score**	**Interpretation**
Students' perception of learning	0-12	Very poor
	13-24	Teaching is viewed negatively
	25-36	A more positive approach
	37-48	Teaching highly thought of
Students' perception of teachers	0-11	Abysmal
	12-22	In need of some retaining
	23-33	Moving in the right direction
	34-44	Model teachers
Students' academic self perception	0-8	Feeling of total failure
	9-16	Many negative aspects
	17-24	Feeling more on the positive side
	25-32	Confident
Students' perception of atmosphere	0-12	A terrible environment
	13-24	There are many issues that need changing
	25-36	A more positive atmosphere
	37-48	A good feeling overall
Students' social self perception	0-7	Miserable
	8-14	Not a nice place

	15-21	Not too bad
	22-28	Very good socially
	13-24	There are many issues that need changing
	25-36	A more positive atmosphere
	37-48	A good feeling overall
Students' social self perception	0-7	Miserable
	8-14	Not a nice place
	15-21	Not too bad
	22-28	Very good socially
